# Snake venomics of *Bothrops punctatus*, a semiarboreal pitviper species from Antioquia, Colombia

**DOI:** 10.7717/peerj.246

**Published:** 2014-01-22

**Authors:** Maritza Fernández Culma, Jaime Andrés Pereañez, Vitelbina Núñez Rangel, Bruno Lomonte

**Affiliations:** 1 Programa de Ofidismo/Escorpionismo, Universidad de Antioquia UdeA, Medellín, Colombia; 2 Facultad de Química Farmacéutica, Universidad de Antioquia UdeA, Medellín, Colombia; 3 Escuela de Microbiología, Universidad de Antioquia UdeA, Medellín, Colombia; 4 Instituto Clodomiro Picado, Facultad de Microbiología, Universidad de Costa Rica, San José, Costa Rica

**Keywords:** Snake venom, Viperidae, Proteomics, *Bothrops punctatus*

## Abstract

*Bothrops punctatus* is an endangered, semi-arboreal pitviper species distributed in Panamá, Colombia, and Ecuador, whose venom is poorly characterized. In the present work, the protein composition of this venom was profiled using the ‘snake venomics’ analytical strategy. Decomplexation of the crude venom by RP-HPLC and SDS-PAGE, followed by tandem mass spectrometry of tryptic digests, showed that it consists of proteins assigned to at least nine snake toxin families. Metalloproteinases are predominant in this secretion (41.5% of the total proteins), followed by C-type lectin/lectin-like proteins (16.7%), bradykinin-potentiating peptides (10.7%), phospholipases A_2_ (93%), serine proteinases (5.4%), disintegrins (38%), L-amino acid oxidases (3.1%), vascular endothelial growth factors (17%), and cysteine-rich secretory proteins (1.2%). Altogether, 6.6% of the proteins were not identified. *In vitro*, the venom exhibited proteolytic, phospholipase A_2_, and L-amino acid oxidase activities, as well as angiotensin-converting enzyme (ACE)-inhibitory activity, in agreement with the obtained proteomic profile. Cytotoxic activity on murine C2C12 myoblasts was negative, suggesting that the majority of venom phospholipases A_2_ likely belong to the acidic type, which often lack major toxic effects. The protein composition of *B. punctatus* venom shows a good correlation with toxic activities here and previously reported, and adds further data in support of the wide diversity of strategies that have evolved in snake venoms to subdue prey, as increasingly being revealed by proteomic analyses.

## Introduction

The Chocoan forest lancehead, *Bothrops punctatus*, known in Colombia as ‘rabo de chucha’, is a large semi-arboreal pitviper, ranging from 1.0 to 1.5 m in length. (Campbell & Lamar, 2004) described its distribution from the Pacific foothills and coastal plain of eastern Panamá through western Colombia to northwestern Ecuador, with an altitudinal range between 135 and 2300 m. In Colombia (Daza, Quintana & Otero, 2005) reported the occurence of *B. punctatus* in the Cauca and Magdalena river basins of Antioquia to eastern Chocó. Although *Bothrops* species are clearly predominant in the epidemiology of snakebite accidents occuring in Colombia (Otero, 1994; Paredes, 2012), published reports of proven envenomings caused by *B. punctatus* appear to be rare. The protein composition of the venom of this species has not been investigated, although at least two reports characterized its toxicological properties, in comparative studies of snake venoms from Colombia (Otero *et al.*, 1992) and Ecuador (Kuch *et al.*, 1996), respectively. The lethal potency of this venom to mice was highest among the different *Bothrops* venoms analyzed in these two studies, being only second to that of *Crotalus durissus terrificus* venom (Otero *et al.*, 1992; Kuch *et al.*, 1996). Due to the lack of knowledge on the venom composition of *B. punctata*, this work aimed at characterizing its proteomic profile using the ‘snake venomics’ analytical strategy (Calvete, Juárez & Sanz , 2007; Calvete , 2011), in combination with the assessment of its enzymatic or toxic activities *in vitro*.

## Methods

### Venom

Venom was obtained from two adult *Bothrops punctatus* specimens collected in the eastern region of the Department of Antioquia, and kept in captivity at the Serpentarium of Universidad de Antioquia, Medellín, Colombia, under institutional, permission for Programa de Ofidismo/Escorpionismo. Venom samples were centrifuged to remove debris, pooled, lyophilized and stored at −20°C. In some functional assays, pooled venom obtained from more than 30 specimens of *Bothrops asper*, collected in the Departments of Antioquia and Chocó, was included for comparative purposes.

### Proteomic profiling

For reverse-phase (RP) HPLC separations, 2.5 mg of venom was dissolved in 200 *μ*L of water containing 0.1% trifluoroacetic acid (TFA; solution A), centrifuged for 5 min at 15,000 × g, and loaded on a C_18_ column (250× 4.6 mm, 5 *μ*m particle; Teknokroma) using an Agilent 1200 chromatograph with monitoring at 215 nm. Elution was performed at 1 mL/min by applying a gradient towards solution B (acetonitrile, containing 0.1% TFA), as follows: 0% B for 5 min, 0–15% B over 10 min, 15–45% B over 60 min, 45–70% B over 10 min, and 70% B over 9 min (Lomonte *et al.*, 2014). Fractions were collected manually, dried in a vacuum centrifuge, and further separated by SDS-PAGE under reducing or non-reducing conditions, using 12% gels. Protein bands were excised from Coomassie blue R-250-stained gels and subjected to reduction with dithiothreitol (10 mM) and alkylation with iodoacetamide (50 mM), followed by in-gel digestion with sequencing grade bovine trypsin (in 25 mM ammonium bicarbonate, 10% acetonitrile) overnight on an automated processor (ProGest Digilab), according to the manufacturer. The resulting peptide mixtures were analyzed by MALDI-TOF-TOF mass spectrometry on an Applied Biosystems 4800-Plus instrument. Peptides were mixed with an equal volume of saturated *α*-CHCA matrix (in 50% acetonitrile, 0.1% TFA), spotted (1 *μ*L) onto Opti-TOF 384-well plates, dried, and analyzed in positive reflector mode. Spectra were acquired using a laser intensity of 3000 and 1500 shots/spectrum, using as external standards CalMix-5 (ABSciex) spotted on the same plate. Up to 10 precursor peaks from each MS spectrum were selected for automated collision-induced dissociation MS/MS spectra acquisition at 2 kV, in positive mode (500 shots/spectrum, laser intensity of 3000). The resulting spectra were analyzed using ProteinPilot v.4 (ABSciex) against the UniProt/SwissProt database using the Paragon®; algorithm at a confidence level of ≥95%, for the assignment of proteins to known families. Few peptide sequences with lower confidence scores were manually searched using BLAST (http://blast.ncbi.nlm.nih.gov). Finally, the relative abundance of each protein (% of total venom proteins) was estimated by integration of the peak signals at 215 nm, using Chem Station B.04.01 (Agilent). When a peak from HPLC contained two or more SDS-PAGE bands, their relative distribution was estimated by densitometry using the Image Lab v.2.0 software (Bio-Rad) (Calvete , 2011).

### Venom activities

#### Phospholipase A_**2**_ activity

Venom phospholipase A_2_ (PLA_2_) activity was determined on the monodisperse synthetic substrate 4-nitro-3-octanoyl-benzoic acid (NOBA) (Holzer & Mackessy, 1996), in triplicate wells of microplates. Twenty *μ*L of venom solutions, containing 20 *μ*g protein, were mixed with 20 *μ*L of water, 200 *μ*L of 10 mM Tris, 10 mM CaCl_2_, 100 mM NaCl, pH 8.0 buffer, and 20 *μ*L of NOBA (0.32 mM final concentration). Plates were incubated at 37°C, and the change in absorbance at 425 nm was recorded after 20 min in a microplate reader (Awareness Technology).

### Proteolytic activity

Proteolysis was determined upon azocasein (Sigma-Aldrich) as described by (Wang, Shih & Huang, 2004). Twenty *μ*g of venoms were diluted in 20 *μ*L of 25 mM Tris, 0.15 M NaCl, 5 mM CaCl_2_, pH 7.4 buffer, added to 100 *μ*L of azocasein (10 mg/mL) and incubated for 90 min at 37°C. The reaction was stopped by adding 200 *μ*L of 5% trichloroacetic acid. After centrifugation, 100 *μ*L of supernatants were mixed with an equal volume of 0.5 M NaOH, and absorbances were recorded at 450 nm. Experiments were carried out in triplicate.

### L-amino acid oxidase activity

L-amino acid oxidase (LAAO) activity was determined by adding various concentrations of venom (2.5–20 *μ*g) in 10 *μ*L of water to 90 *μ*L of a reaction mixture containing 250 mMl-Leucine, 2 mM *o*-phenylenediamine, and 0.8 U/mL horseradish peroxidase, in 50 mM Tris, pH 8.0 buffer, in triplicate wells of a microplate (Kishimoto & Takahashi, 2001). After incubation at 37°C for 60 min, the reaction was stopped with 50 *μ*L of 2 M H_2_SO_4_, and absorbances were recorded at 492 nm.

### Cytotoxic activity

Cytotoxic activity was assayed on murine skeletal muscle C2C12 myoblasts (ATCC CRL-1772) as described by (Lomonte *et al.*, 1999) Venom (40 *μ*g) was diluted in assay medium (Dulbecco’s Modified Eagle’s Medium [DMEM] supplemented with 1% fetal calf serum [FCS]), and added to subconfluent cell monolayers in 96-well plates, in 150 *μ*L, after removal of growth medium (DMEM with 10% FCS). Controls for 0 and 100% toxicity consisted of assay medium and 0.1% Triton X-100 diluted in assay medium, respectively. After 3 h at 37°C, a supernatant aliquot was collected to determine the lactic dehydrogenase (LDH; EC 1.1.1.27) activity released from damaged cells, using a kinetic assay (Wiener LDH-P UV). Experiments were carried out in triplicate.

### ACE inhibitory activity

The angiotensin-converting enzyme (ACE) inhibitory activity of fraction 4 from the HPLC separation (see Table 1), which was identified as a bradykinin-potentiating peptide-like component, was assayed by the method of (Cushman & Cheung, 1971) with some modifications (Kim *et al.*, 1999). Various concentrations of the fraction, diluted in 20 *μ*L, were added to 100 *μ*L of 10 mM N-hippuryl-His-Leu substrate diluted in 2 mM potassium phosphate, 0.6 M NaCl, pH 8.3 buffer, and 5 mU of ACE (EC 3.4.15.1; 5.1 UI/mg) diluted in 50% glycerol. The reaction was incubated at 37°C for 30 min, and stopped by adding 200 *μ*L of 1 NHCl. The produced hippuric acid was extracted by vigorous stirring for 10 s, followed by the addition of 600 *μ*L of ethyl acetate, and centrifugation for 10 min at 4000 × g. An aliquot of 500 *μ*L of organic phase was dried at 95°C for 10 min. The residue was dissolved in 1 mL of water and, after stirring, the absorbance was measured at 228 nm. The percentage of ACE inhibition (% ACEi) was determined using the following formula; % ACEi = (Abs Control–Abs sample)/(Abs control–Abs blank). Control absorbance corresponded to hippuric acid formed after the action of ACE, while blank absorbance was enzyme without substrate.

## Statistical analyses

The significance of differences between means was assessed by ANOVA, followed by Dunnett’s test, when several experimental groups were compared with the control group, or by Student’s t-test, when two groups were compared. Differences were considered significant if  *p* < 0.05.

## Results and Discussion

*B. punctatus* has been included in the ‘red list’, a report categorizing conservation status, as a threatened species (Carrillo *et al.*, 2005). Very scarce information on its venom is available in the literature. In comparative studies of snake venoms from Colombia (Otero *et al.*, 1992) and Ecuador (Kuch *et al.*, 1996), respectively, this venom was found to induce local effects such as hemorrhage, edema, and myonecrosis, as well as systemic alterations such as defibrination, in similarity to venoms from other *Bothrops* species. Developments in proteomic techniques have brought new possibilities to examine the detailed toxin composition of snake venoms, increasing knowledge on their evolution, toxicological properties, and correlation with clinical features of envenomings (Calvete, Juárez & Sanz , 2007; Calvete , 2013; Fox & Serrano, 2008; Valente *et al.*, 2009; Ohler *et al.*, 2010; Georgieva *et al.*, 2010). Therefore, the venom of *B. punctatus* was analyzed for the first time using proteomic tools, to gain a deeper understanding on its protein composition and relationships to toxic and enzymatic actions.

**Figure 1:**
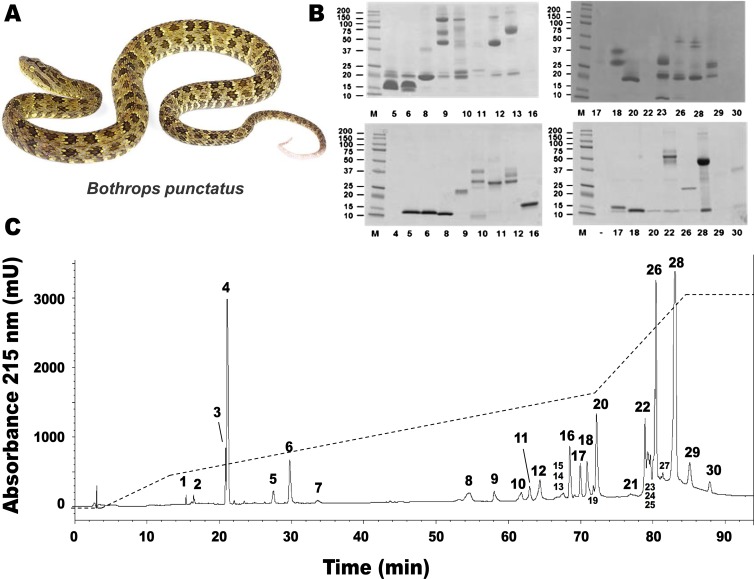
Separation of *Bothrops punctatus* (A) venom proteins by RP-HPLC (C) and SDS-PAGE (B). Venom was fractionated on a C_18_ column (C) by applying an acetonitrile gradient from 0 to 70% (dashed line), as described in Methods. Each fraction was analyzed by SDS-PAGE (B) under non-reducing (top gels) or reducing (bottom gels) conditions. Molecular weight markers (M) are indicated in kDa, at the left. Tryptic digests of the excised protein bands were characterized by MALDI-TOF/TOF, as summarized in Table 1. The photograph of *B. punctatus* was obtained with permission from www.tropicalherping.com.

**Figure 2:**
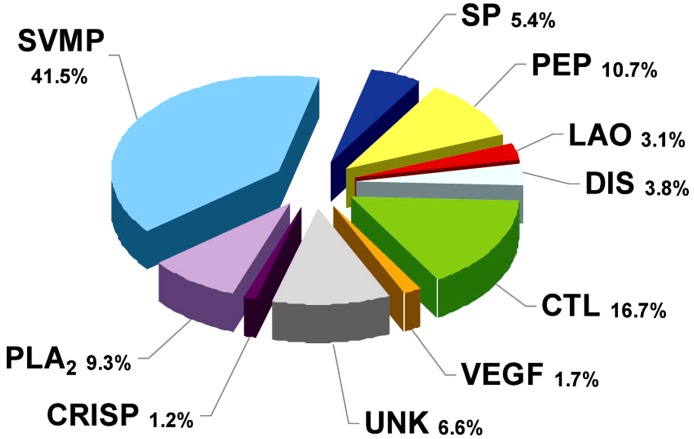
Composition of *Bothrops punctatus* venom according to protein families, expressed as percentages of the total protein content. SP: serine proteinase; PLA_2_: phospholipase A_2_; CRISP: cysteine-rich secretory protein; DIS: disintegrin; PEP: bradykininpotentiating peptide-like (BPP-like); LAO: L-amino acid oxidases; SVMP: metalloproteinase; VEGF: vascular endothelium growth factor; CTL: C-type lectin/lectin-like; UNK: unknown/unidentified.

RP-HPLC of the crude venom resulted in the separation of 30 fractions (Fig. 1C), which were further subjected to SDS-PAGE (Fig. 1B), in-gel digestion of the excised bands, and MALDI-TOF-TOF analysis of the resulting peptides. The amino acid sequences obtained allowed the unambiguous assignment of 29 out of the 37 components analyzed, to known protein families of snake venoms (Table 1). Protein family relative abundances were estimated by integration of the chromatographic areas, combined with gel densitometric scanning. Results showed that the predominant proteins in this secretion are metalloproteinases (41.5%; SVMP), followed by C-type lectin/lectin-like proteins (16.7%; CTL), bradykinin-potentiating peptide-like peptides (10.7%; PEP), phospholipases A_2_ of both the D49 (8.0%) and K49 (1.3%) subtypes (for a combined 9.3%; PLA_2_), serine proteinases (5.4%; SP), disintegrins (38%; DIS), L-amino acid oxidases (3.1%; LAO), vascular endothelial growth factor (1.7%; VEGF), and cysteine-rich secretory proteins (1.2%; CRISP), as summarized in Fig. 2 and Table 1. An estimated 6.6% of the proteins remained unidentified, and owing to the scarcity of the venom, their assignment could not be further pursued.

**Table 1:** Assignment of the RP-HPLC isolated fractions of *Bothrops punctatus* venom to protein families by MALDI-TOF-TOF of selected peptide ions from in-gel trypsin-digested protein bands.

Peak	%	Mass (kDa)	Peptide ion		MS/MS-derived amino acid sequence*	Protein family; ∼ related protein
			m/z	z		
1	0.2		-	-	-	unknown
2	0.3		-	-	-	unknown
3	1.6		-	-	-	unknown
4	10.7	-	967.5	1	ZBWAPVBK	BPP-like; ∼ Q7T1M3
5	0.8	▼ 10	2259.1	1	XARGDDM^*ox*^ DDYCNGXSAGCPR	Disintegrin; ∼ Q7SZD9
			2051.0	1	XRPGABCAEGXCCDBCR	
			2459.0	1	EAGEECDCGTPGNPCCDAATCK	
6	3.0	▼ 10	1902.9	1	GDDMDDYCNGXSAGCPR	Disintegrin; ∼ Q0NZX5
			2243.1	1	XARGDDMDDYCNGXSAGCPR	
			2051.0	1	XRPGABCAEGXCCDBCR	
			2459.1	1	EAGEECDCGTPGNPCCDAATCK	
7	0.3		-	-	-	unknown
8	1.7	▼ 11	2062.0	1	CGGCCTDESXECTATGBR	VEGF; ∼ Q90X23
			3134.9	1	ETXVSXXEEHPDEVSHXFRPSCVTAXR	
9	1.2	▼ 22 ■ 18	2526.1	1	SGPPCGDCPSACDNGXCTNPCTK	CRISP; ∼ Q7ZT99
			1537.8	1	MEWYPEAAANAER	
			1828.9	1	YFYVCBYCPAGNMR	
10a	0.4	▼ 38	1561.9	1	SVPNDDEEXRYPK	Serine proteinase; ∼ Q5W960
10b	0.2	▼ 29 ■ 28	1206.8	1	XMGWGTXSPTK	Serine proteinase; ∼ Q072L6
			1683.2	1	TYTBWDBDXMXXR	
			2534.5	1	VSYPDVPHCANXNXXDYEVCR	
			1069.8	1	FXVAXYTSR	
			1512.8	1	VXGGDECNXNEHR	
			3387.8	1	DSCBGDSGGPXXCNGBFBGXXSWGVHPCGBR	
10c	0.3	▼ 12 ■ 22	-	-	-	unknown
11	1.5	▼ 28 ■ 20	1288.7	1	NFBMBXGVHSK	Serine proteinase; ∼ Q072L6
			1190.7	1	XMGWGTXSPTK	
			2305.4	1	AAYPWBPVSSTTXCAGXXBGGK	
			1140.6	1	VSDYTEWXK	
			2477.5	1	VSNSEHXAPXSXPSSPPSVGSVCR	
			2477.4	1	VXGGDECNXNEHR	
12a	1.8	▼ 35	1083.7	1	FXAFXYPGR	Serine proteinase; ∼ Q6IWF1
12b	0.4	▼ 29 ■ 22	1517.9	1	NDDAXDBDXMXVR	Serine proteinase; ∼ Q5W959
			1499.8	1	VVGGDECNXNEHR	
			2294.3	1	TNPDVPHCANXNXXDDAVCR	
			1279.7	1	AAYPEXPAEYR	
			2889.7	1	XDSPVSNSEHXAPXSXPSSPPSVGSVCR	
			1083.7	1	FXAFXYPGR	
13-15	0.8		-	-	-	unknown
16	3.1	▼ 16 ■ 16	1505.7	1	CCFVHDCCYGK	Phospholipase A_2_, D49; ∼ P86389
			934.6	1	YWFYGAK	
			1966.1	1	YXSYGCYCGWGGXGBPK	
			2064.1	1	DATDRCCFVHDCCYGK	
			2027.2	1	DNBDTYDXBYWFYGAK	
			2626.4	1	XDXYTYSBETGDXVCGGDDPCBK	
			1786.0	1	BXCECDRVAATCFR	
17a	0.4	▼ 14 ■ 21	1928.9	1	DCPPDWSSYEGHCYR	C-type lectin/lectin-like; ∼ P22030
17b	1.7	▼ 15 ■ 16	2027.1	1	DNBDTYDXBYWFYGAK	Phospholipase A_2_, D49; ∼ C9DPL5
17c	0.4	■ 13	1720.8	1	E^*pa*^ NGDVVCGGDDPCBK	Phospholipase A_2_, D49; ∼ P86389
			1505.7	1	CCFVHDCCYGK	
			2064.0	1	DATDRCCFVHDCCYGK	
18	2.8	▼ 13	2064.0	1	DATDRCCFVHDCCYGK	Phospholipase A_2_, D49; ∼ Q9I968
19	0.3		-	-	-	unknown
20	6.2	▼ 13 ■ 19	1928.9	1	DCPSDWSPYEGHCYR	C-type lectin/lectin-like; ∼ Q9PS06
21	0.8		-	-	-	unknown
22a	0.9	■ 120	1537.8	1	ACSNGBCVDVNRAS	Metalloproteinase; ∼ Q8AWI5
			1269.7	1	SAECTDRFBR	
22b	3.1	▼ 53 ■ 48	3185.9	1	VVXVGAGMSGXSAAYVXANAGHBVTVXEASER	L-amino acid oxidase; ∼ Q6TGQ9
			2605.5	1	BFGXBXNEFSBENENAWYFXK	
			2271.3	1	XYFAGEYTABAHGWXDSTXK	
			1388.8	1	BFWEDDGXHGGK	
			1352.8	1	SAGBXYEESXBK	
22c	0.9	▼ 13	1636.0	1	NXBSSDXYAWXGXR	C-type lectin/lectin-like; ∼ P22029
			1928.9	1	DCPPDWSSYEGHCYR	
23-25a	1.3	▼ 13	1533.7	1	SYGAYGCNCGVXGR	Phospholipase A_2_, K49; ∼ Q9PVE3
23-25b	1.1	▼ 28, ■ 20	1279.7	1	AAYPEXPAEYR	Serine proteinase; ∼ Q5W959
			14.997	1	VVGGDECNXNEHR	
			2294.1	1	TNPDVPHCANXNXXDDAVCR	
23-25c	0.9	▼ 13, ■ 19	1635.8	1	NXBSSDXYAWXGXR	C-type lectin/lectin-like; ∼ P22029
26	14.4	▼ 23 ■ 42	2040.2	1	YXYXDXXXTGVEXWSNK	Metalloproteinase; ∼ P86976
			1114.6	1	XHBMVNXMK	
			2257.3	1	DXXNVBPAAPBTXDSFGEWR	
			1828.0	1	YVEXFXVVDHGMFMK	
27	2.0		-	-	-	unknown
28a	18.3	▼ 46 ■ 42	1552.7	1	VCSNGHCVDVATAY	Metalloproteinase; ∼ Q8QG88
			2953.3	1	ASM^*ox*^ SECDPAEHCTGBSSECPADVFHK	
			2154.2	1	XTVBPDVDYTXNSFAEWR	
28b	2.1	■ 21	3261.7	1	TDXVSPPVCGNYFVEVGEDCDCGSPATCR	Metalloproteinase; ∼ O93517
			1457.0	1	XVXVADYXM^*ox*^ FXK	
28c	6.2	▼ 14	1635.9	1	NXBSSDXYAWXGXR	C-type lectin-like; ∼ P22029
			1193.6	1	TTDNBWWSR	
29a	3.2	▼ 46	2154.2	1	XTVBPDVDYTXNSFAEWR	Metalloproteinase; ∼ Q8QG88
			1609.9	1	XYEXVNTXNVXYR	
			1775.0	1	YVEFFXVVDBGMVTK	
29b	2.1	▼ 14	992.5	1	MNWADAER	C-type lectin/lectin-like; ∼ M1V359
			1928.8	1	DCPPDWSSYEGHCYR	
			1842.9	1	MNWADAERFCSEQAK	
30	2.6	▼ 38	1327.8	1	YXEXVXVADHR	Metalloproteinase; ∼ Q8AWX7

* Cysteine residues determined in MS/MS analyses are carbamidomethylated. X: Leu/Ile; B: Lys/Gln; ^*ox*^: oxidized; ^*pa*^: propionamide; ▼: reduced, or ■: non-reduced SDS-PAGE mass estimations, in kDa. Abbreviations for protein families as in Fig. 2.

**Figure 3:**
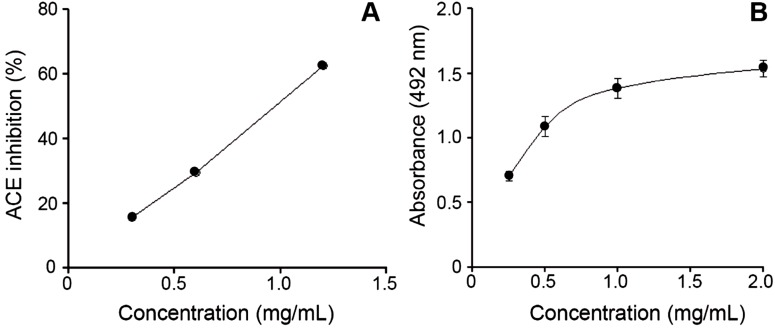
*Bothrops punctatus* venom activities. (A) Inhibition of angiotensin-converting enzyme (ACE) by peak 4 of *B. punctatus* venom, identified as a BPP-like peptide (Table 1). Each point represents the mean ± SD of three replicates. (B) L-amino acid oxidase activity of *B. punctatus* venom. Each point represents the mean ± SD of three replicates.

A recent phylogenetic analysis of the genus *Bothrops* (*sensu lato*) by (Fenwick *et al.*, 2009) grouped *B. punctatus* within the same clade as *Bothrops atrox* and *Bothrops asper*. Since the proteomic profile of the venoms of the latter two species has been reported (Núñez *et al.*, 2009; Alape-Girón *et al.*, 2008), a comparison of their venom compositions, together with those of two other pitviper species distributed in Colombia, *Bothrops ayerbei* (Mora-Obando *et al.*, 2014) and *Bothriechis schlegelii* (Lomonte *et al.*, 2008), was compiled (Table 2). Venoms from these five species have been analyzed by the same methodological strategy, therefore allowing reliable comparisons. The composition of *B. punctatus* venom resembles that of the other *Bothrops* species listed in Table 2 only in terms of their high content of metalloproteinases (41.5–53.7%), but overall, its composition departs from the relative protein abundances observed in any of the other four pitvipers. The high proportion of CTL proteins in *B. punctatus* is of note, doubling the abundance observed in *B. atrox*, and close to that of *B. ayerbei*, while in contrast such proteins are expressed only in trace amounts in *B. asper*, and have not been detected in *B. schlegelii* (Table 2). Further, *B. punctatus* venom presents a modest amount of VEGF (1.7%), which has not been found in any of the venoms listed in Table 2. Similar to the venom of the arboreal snake *B. schlegelii*, but also with the terrestrial species *B. ayerbei*, the venom of *B. punctatus* presents a high content of BPP-like peptides, strikingly differing from *B. asper* and *B. atrox* venoms in this regard. The possible trophic relevance of these vasoactive peptides among viperids remains elusive, and no clear correlations with prey types or habitats have been disclosed thus far. BPPs are oligopeptides of 5–14 amino acid residues, rich in proline residues and often presenting a pyroglutamate residue, which display bradykinin-potentiating activity. Their pharmacological effect is related to the inhibition of angiotensin I-converting enzyme (ACE) (Ianzer *et al.*, 2007). Peak 4 of the HPLC separation of *B. punctatus* venom components (Fig. 1C) was identified as a BPP (Table 1), and its inhibitory activity on ACE was confirmed showing a half-maximal inhibition of this enzyme at 0.9 mg/mL (Fig. 3A). Interest in snake venom BPPs stems from their potential in the development of hypotensive drugs, as exemplified by Captopril®;. Overall, the comparison of *B. punctatus* venom with those of other pitvipers distributed in Colombia (Table 2) highlights the remarkable divergence of compositional profiles that have arisen through the evolution and diversification of snakes (Casewell *et al.*, 2013).

**Table 2:** Comparison of the venom composition of *Bothrops punctatus* with venoms from pitviper species distributed in Colombia*

Protein family	Snake species				
2-6	*Bothrops punctatus* a	*Bothrops atrox* b	*Bothrops asper* c	*Bothriechis schlegelii* d	*Bothrops ayerbei* e
Metalloproteinase	41.5	48.5	44.0	17.7	53.7
Phospholipase A_2_	9.3	24.0	45.1	43.8	0.7
Serine proteinase	5.4	10.9	10.9	5.8	9.3
BPP-like	10.7	0.3	-	13.4	8.3
CRISP	1.2	2.6	0.1	2.1	1.1
C-type lectin/lectin-like	16.7	7.1	0.5	-	10.1
VEGF	1.7	-	-	-	-
L-amino acid oxidase	3.1	4.7	4.6	8.9	3.3
Disintegrin	3.8	1.7	1.4	-	2.3
Kazal type inhibitor	-	-	-	8.3	-
Phosphodiesterase	-	-	-	-	0.7
Nerve growth factor	-	-	-	-	0.1
unknown	6.6	-	-	-	1.7
**Number of families**	**9**	**8**	**7**	**7**	

* Although *B. asper* and *B. schlegelii* are found in Colombia, data correspond to venoms from specimens found in Costa Rica.

a Present work.

b (Núñez *et al.*, 2009).

c (Alape-Girón *et al.*, 2008), specimens of Pacific versant.

d (Lomonte *et al.*, 2008).

e (Mora-Obando *et al.*, 2014).

**Figure 4:**
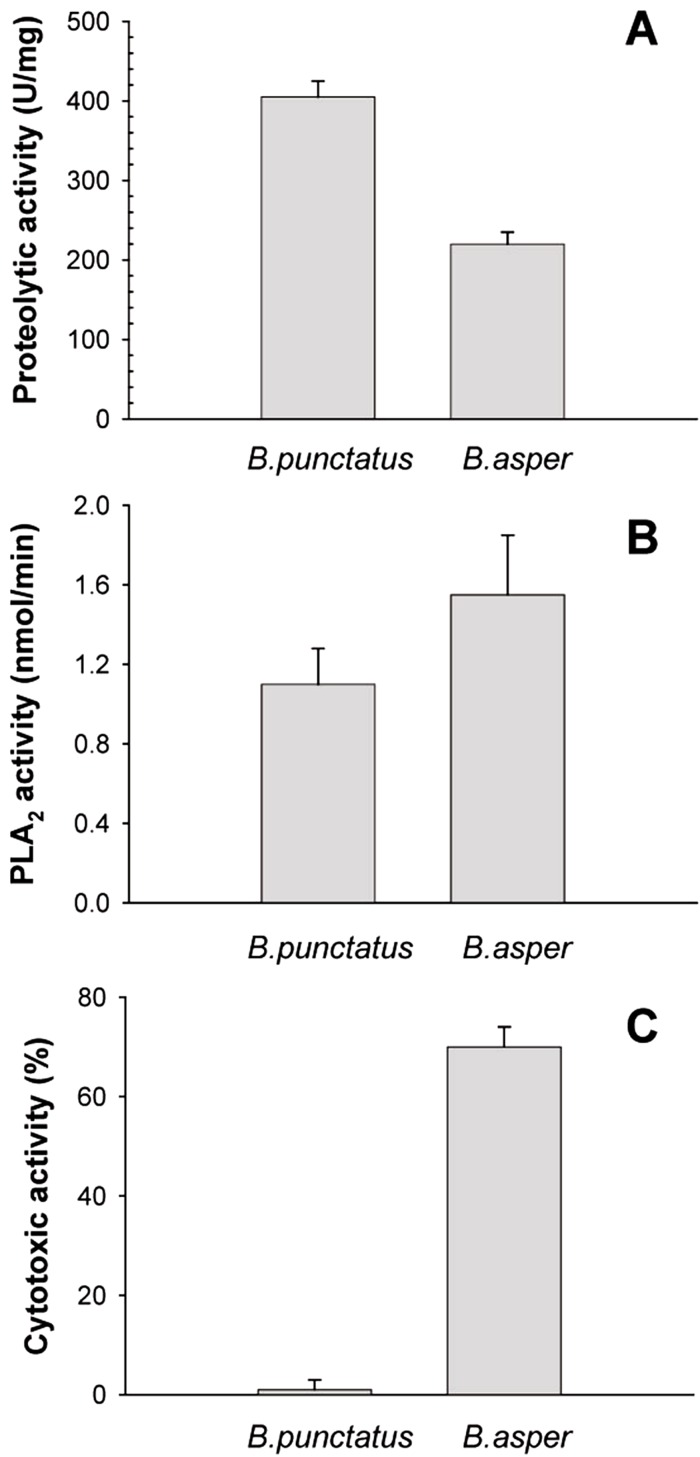
Proteolytic (A), phospholipase A_2_ (B), and cytotoxic (C) activities of *Bothrops punctatus* venom, compared to the venom of *Bothrops asper* Proteolytic activity was determined on azocasein, using 20 *μ*g of each venom. Phospholipase A_2_ activity was determined on 4-nitro-3-octanoyloxy-benzoic acid, using 20 *μ*g of each venom Cytotoxic activity was determined on C2C12 murine myoblasts, using 40 *μ*g of each venom, as described in Methods. Bars represent mean ± SD of three replicates. For each activity, differences between the two venoms were significant (*p* < 0.05).

The protein composition of *B. punctatus* venom correlates with the enzymatic activities assayed, as well as with those described in earlier studies (Otero *et al.*, 1992; Kuch *et al.*, 1996). L-amino acid oxidase (Fig. 3C), proteolytic (Fig. 4A), and PLA_2_ (Fig. 4B) activities of this venom were corroborated. Interestingly, its proteolytic activity was higher than that of *B. asper* venom (Fig. 4A), and this might be related to the stronger hemorrhagic potency that was reported for *B. punctatus* venom in comparison to *B. asper* venom (Otero *et al.*, 1992). Hemorrhage induced by viperid venoms is mainly dependent on the proteolytic action of SVMPs upon the microvasculature and its extracellular matrix support (Bjarnason & Fox, 1994; Gutiérrez *et al.*, 2005), and this effect can be enhanced by venom components affecting haemostasis, such as procoagulant SPs with thrombin-like activity, or some CTL components and disintegrins that potently interfere with platelets, among others (Gutiérrez, Escalante & Rucavado, 2009; Calvete *et al.*, 2005). Considering that the proportion of SVMPs is lower in *B. punctatus* than in *B. asper* venom (Table 2), the higher hemorrhagic action reported for the former (Otero *et al.*, 1992) suggests that its abundant CTL components (16.7%) might include toxins that affect platelets, a hypothesis that deserves future investigation. On the other hand, the PLA_2_ activity of *B. punctatus* venom was lower than that of *B. asper* (Fig. 4B), in agreement with their corresponding relative contents of these enzymes (Table 2). However, a major contrast was evidenced in the cytotoxic activity of these two venoms upon myogenic cells in culture, *B. punctatus* being essentially devoid of this effect, while *B. asper* causing overt cytolysis and LDH release under identical conditions (Fig. 4C). Since cytolysis of myogenic cells, an *in vitro* correlate for *in vivo* myotoxicity (Lomonte *et al.*, 1999), has been shown to be mediated mainly by basic PLA_2_s in the case of viperid venoms (Gutiérrez & Lomonte, 1995; Lomonte & Rangel, 2012), this finding anticipates that the catalytically active (D49) PLA_2_s present in *B. punctatus* venom are likely to belong to the acidic type of these enzymes, which despite frequently having higher enzymatic activity than their basic counterparts, usually display very low, or even no toxicity (Fernández *et al.*, 2010; Van der Laat *et al.*, 2013). In contrast, the venom of *B. asper* is rich in basic D49 and K49 PLA_2_s/PLA_2_ homologues with strong cytolytic and myotoxic effects (Angulo & Lomonte, 2005, 2009) that would explain the present findings. Although at least one PLA_2_ component of *B. punctatus* venom was shown to belong to the K49 type of catalytically-inactive, basic PLA_2_ homologues (fraction 23–25a; Table 1), its low abundance (1.3%) in the venom would be in agreement with the observed lack of cytotoxicity (Fig. 4C).

In summary, the general compositional profile of *B. punctatus* venom was obtained through the analytical strategy known as ‘snake venomics’. The present data add to the growing body of knowledge on the remarkable diversity of compositional strategies in snake venom ‘cocktails’, in spite of the reduced number of gene families that encode their proteins/toxins (Casewell *et al.*, 2013; Calvete , 2013). Due to the key adaptive role of venoms, this knowledge, in combination with toxicological, ecological, and natural history information, could lead to a deeper understanding of the evolutionary trends and selective advantages conferred by particular venom compositions in the divergence of snakes. In addition, compositional data may offer a more comprehensive basis to foresee the features of envenomings by this pitviper species, largely unreported in the literature.
